# Super-resolution optical telescopes with local light diffraction shrinkage

**DOI:** 10.1038/srep18485

**Published:** 2015-12-18

**Authors:** Changtao Wang, Dongliang Tang, Yanqin Wang, Zeyu Zhao, Jiong Wang, Mingbo Pu, Yudong Zhang, Wei Yan, Ping Gao, Xiangang Luo

**Affiliations:** 1 State Key Laboratory of Optical Technologies on Nano-Fabrication and Micro-Engineering, Institute of Optics and Electronics, Chinese Academy of Sciences, P.O. Box 350, Chengdu 610209, China; 2 University of Chinese Academy of Sciences, Beijing 100049, China

## Abstract

Suffering from giant size of objective lenses and infeasible manipulations of distant targets, telescopes could not seek helps from present super-resolution imaging, such as scanning near-field optical microscopy, perfect lens and stimulated emission depletion microscopy. In this paper, local light diffraction shrinkage associated with optical super-oscillatory phenomenon is proposed for real-time and optically restoring super-resolution imaging information in a telescope system. It is found that fine target features concealed in diffraction-limited optical images of a telescope could be observed in a small local field of view, benefiting from a relayed metasurface-based super-oscillatory imaging optics in which some local Fourier components beyond the cut-off frequency of telescope could be restored. As experimental examples, a minimal resolution to 0.55 of Rayleigh criterion is obtained, and imaging complex targets and large targets by superimposing multiple local fields of views are demonstrated as well. This investigation provides an access for real-time, incoherent and super-resolution telescopes without the manipulation of distant targets. More importantly, it gives counterintuitive evidence to the common knowledge that relayed optics could not deliver more imaging details than objective systems.

Since it was invented about four hundred years ago, the optical telescope has always been the important tool for applications requiring optical information of targets long-distance away, like astronomy observation, optical surveillance and remote sensing. Suffering from the diffraction of light, the resolution of a telescope is theoretically limited by the diameter *D* of the objective aperture and light wavelength *λ*, in some form like Rayleigh criterion 1.22*λ/D*. This resolution obstacle has been fully realized for centuries and extensively interpreted with the uncertainty principle from the viewpoint of quantum mechanics^1^,^2^. Consequently, the main access to enhance the resolution of telescope relies on the increase of the aperture size of an objective lens, besides some complementary but important efforts, including adaptive optics for air distortion compensation^3^ and recording devices with high sensitivity and density^4^. On the other hand, to reduce the complexities and challenges to build an ultra-large telescope, some innovative concepts are proposed, such as Fourier transform telescope^5^, aperture-synthesis^6^ and banked radio telescopes^7^. Unfortunately, those methods still suffer from the diffraction limit and are usually restricted to specific applications due to the concerns for the posterior data processing and active illumination.

Recently, some novel concepts are proposed and deliver revolutionary ideas for super-resolution optics. Perfect lens composed of a negative refractive index (NRI) metamaterial slab was proved in theory to be capable of imaging infinite small features of targets^8^. This idea was further extended to the superlens and hyperlens, which was applied in nano-lithography and helped to improve the resolution of an optical microscope^9^,^10^,^11^,^12^,^13^,^14^,^15^,^16^. Based on fluorescence radiation manipulations, some super-resolution microscopes, including stimulated emission depletion microscopy (STED)^17^, stochastic optical reconstruction microscopy (STORM)^18^ and photoactivated localization microscopy (PALM)^19^, delivered impressive achievements in observing nanoscale fluorescence targets. Recently, it was experimentally demonstrated that a super-oscillatory lens (SOL) generating a sub-wavelength focusing spot could be applied for a super-resolution microscope in confocal scanning manner with coherent light illumination^20^. In contrast to the great interests and achievements in super-resolution optics, especially in the applications of microscopes and nano-lithography, few efforts are made to break the diffraction barrier of telescopes. This could be mainly attributed to the fact that remote objects, like celestial targets, would not be pragmatically accessible for artificial radiation manipulation and the great size of telescopes hamper the possibility of super-resolution lenses with metamaterial slabs.

In this paper, we would like to present a real-time, free of posterior data processing and incoherent super-resolution telescope (SRT) and proof-of-principle experimental results. The SRT system benefits from the shrinkable lateral full width of local point spread function (LPSF) beyond the diffraction limit. This feature, from the viewpoint of defined local optical transfer function (LOTF), enables higher local Fourier frequency components surpassing the cut-off frequency determined by the finite aperture size of the objective lens. Real-time super-resolution images are optically restored in a local field of view from the diffraction-limited optical images generated by an objective lens, without the need of posterior data processing or complex radiation manipulation of targets. In our demonstrative experiments, a greatly reduced LPSF with the lateral full width of about 0.5 times that of Airy spot is obtained, yielding a minimal resolvable ability to 0.55 of Rayleigh criterion. The method could also be utilized to image complex and large targets by superimposing multiple local fields of views.

## Results

### Principle and design

**Figure 1:**
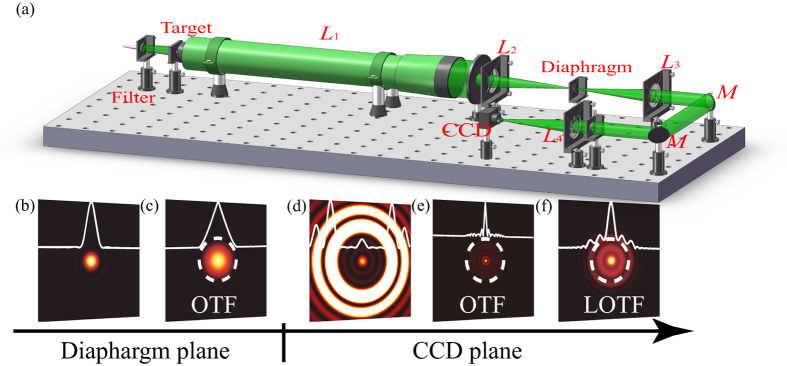
A schematic of SRT system. (**a**) Schematic of the optical measurement setup of a SRT system, including a halogenated lamp, a filter, an optical collimator (*L*_1_), an entrance pupil, an objective lens (*L*_2_) and a 4ƒ system consisting of a field diaphragm, two mirrors (*M*), two focusing lenses (*L*_3_ and *L*_4_), a designed phase plate and a CCD camera. Image courtesy of Dongliang Tang, Institute of Optics and Electronics, Chinese Academy of Sciences. (**b**) Simulated diffraction-limited pattern and (**c**) corresponding OTF at diaphragm plane. (**d**) Simulated sub-diffraction pattern and corresponding (**e**) OTF and (**f**) LOTF at CCD plane. Dashed inset rings are the cut-off frequency of diffraction-limited OTF.

Figure 1a shows the schematic of the incoherent SRT system. The objective lens with finite aperture sizes at the entrance-pupil plane is used to image infinite distant targets, which are positioned at the front focal plane of an optical collimator and illuminated by an incoherent light source. A small region of diffraction-limited optical images, restricted by a field diaphragm at the imaging plane of the objective lens, is relayed through a local diffraction shrinkable optics consisting of a 4ƒ system with a specially designed phase plate at the exit-pupil plane. The generated diffraction pattern is recorded with a CCD.

The point spread function (PSF) of the SRT system, i.e. the diffraction pattern of the phase plate when the light point source is positioned at the central front focal plane of the optical collimator, could be approximately expressed as^21^,^22^


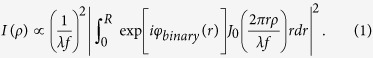


Here ƒ is the focal length of the last lens in the 4ƒ system and R the aperture radius of the exit pupil. The modulation phase function 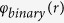 is assumed to be a circular symmetrical binary phase function (0 or π) with finite phase-jump positions and could be optimized by linear programming method to get a sub-diffraction central spot with lateral full width *G* and finite field of view *L*^21^,^22^.

For the case without phase modulation at the wavelength of 532 nm, as shown in Fig. 1b, both the PSF at the imaging plane of the objective lens and that in the SRT system turn to be the Airy spot. Figure 1c shows its spatial Fourier spectrum with limited frequency, determined by the aperture size of the objective lens. Two phase plates with different LPSFs are designed to obtain a local diffraction shrinkable effect for sub-diffraction imaging in this work. The first one (Spot 1), with normalized π-phase-jump positions at *r*_1_ = 0.297, *r*_2_ = 0.594 and *r*_3_ = 0.85, has a 0.6 times the full width of Airy pattern. A smaller focusing spot (Spot 2) with full width of about 0.5 of Airy pattern is optimized with π-phase-jump positions at *r*_1_ = 0.4405 and *r*_2_ = 0.8137. Detailed parameters are presented in Supplementary Table S1.

As clearly seen in Fig. 1d, the diffraction shrinkable phenomenon for Spot 1 could be observed for the central spot with greatly reduced lateral size. Inside the field of view, the maximum intensity *M*_1_ of side-lobes normalized by the central peak intensity is assumed to be small enough to bring no significant influence for super-resolution imaging in this local field of view (*M*_1_ is set as 0.1 in this work). The sub-diffraction pattern defined by Eq. (1) would be attributed to the destructive interference of high and low Fourier components. This feature, we believe, would be the fundamental factor to realize super-resolution imaging optics, especially for those telescope systems which could not seek helps from present near-field and fluorescent super-resolution methods, like a perfect lens with negative refractive index and stimulated emission depletion microscopes.

It should be noted that the access to Fourier components beyond the cut-off frequency would not be possible from the definition of Fourier components in the total focus pattern region, as shown in Fig. 1e of the conventional optical transfer function (OTF). Different conclusions could be obtained as we focus on the small region around the central diffraction shrinkage spot and ignore those great side-lobes outside the field of view. To show this point, the concept of local optical transfer function (LOTF) is proposed with details in the Methods. In the given analyses, the key point is that the Fourier components of LOTF are not contributed from the total diffraction patterns, but only accounts for the sub-diffraction pattern within the local field of view. As a result, local Fourier components beyond the cut-off frequency of conventional OTF could be seen in Fig. 1f. It is reasonable to rely on the tool of LOTF for the SRT imaging analysis, provided that the imaging field is constrained to the local field of view and the side-lobes are neglected in the imaging process. Consequently, a remote target, as the ensemble of a great deal of light spots, could be observed with more details and enhanced resolution, depending on the extent of the diffraction shrinkage.

### Super-resolution imaging in SRT system

**Figure 2:**
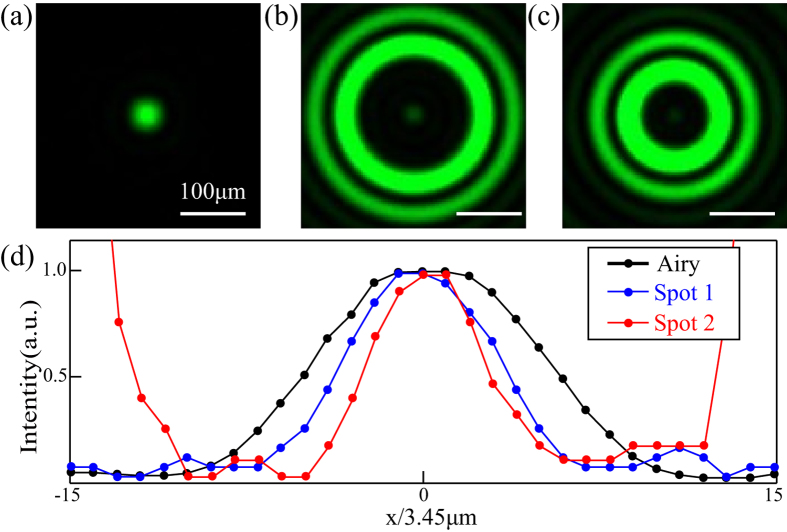
LPSF of the SRT system. (**a–c**) Experimental patterns at CCD plane for Airy, Spot 1 and Spot 2, respectively. (**d**) Corresponding field distributions along horizontal line across the center.

In the experiment, as the incoherent light is incident on the 20 μm hole at the front focal plane of *L*_1_, three spots with different full width sizes at the CCD plane were measured to be 82.8 μm, 51.75 μm and 41.4 μm for Airy spot, Spots 1 and 2, respectively. The two diffraction shrinkable spots are about 0.63 and 0.5 times that of Airy spot, as shown in Fig. 2. Detailed experimental parameters are presented in Supplementary Table S1.

**Figure 3:**
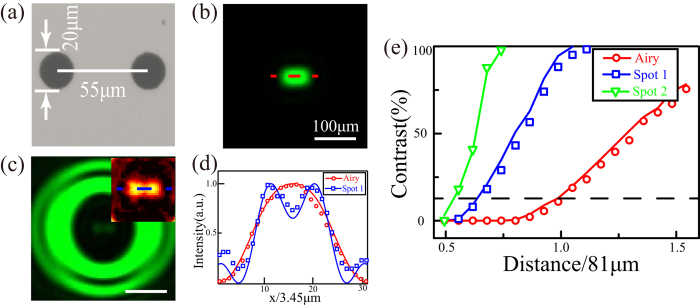
Resolvable ability of the SRT system. (**a**) Microscope image of a two-hole target. Experimental (**b**) diffraction-limited imaging pattern and (**c**) super-resolution imaging pattern with Spot 1. False-color inset shows the magnified central field distribution. (**d**) Experimental (symbolic lines) and simulated field (solid lines) distributions along horizontal lines in (**b**,**c**). (**e**) Experimental (symbolic lines) and simulated (solid lines) imaging contrast for variant center-to-center distances of two-hole targets. Dashed line indicates the resolvable contrast of Rayleigh criterion.

To demonstrate the resolvable ability of the SRT, two transparent holes with 20 μm diameter and 55 μm center-to-center distance, corresponding to 0.68 of equivalent Rayleigh criterion distance 81.13 μm (see Supplementary Information for details), are used for super-resolution imaging evaluation (Fig. 3a). As expected, the two holes are completely unresolved at the imaging plane of the SRT’s objective lens (Fig. 3b). The information seems to be lost forever at the entrance of the objective lens, from the viewpoint of conventional Fourier optics^23^. However, at the CCD plane of the relayed optics with Spot 1, the two holes are clearly distinguished with resolution beyond the Rayleigh criterion (Fig. 3c). The measured result in Fig. 3d shows good agreement with the simulation, performed by convolving the objective function with LPSF. Figure 3e plots the experimental and simulated imaging contrasts for variant center-to-center distances of two holes. Both the imaging resolution and contrast could be remarkably improved in the SRT system with Spots 1 and 2. The minimal resolvable distance with Spot 2 reaches to about 0.55 of equivalent Rayleigh criterion distance.

Definitely, the imaging results indicate that the proposed SRT method is different from the inverse filter method^24^, in which incoherent imaging is restricted and image information beyond the diffraction limit could not be restored in theory. The diffraction shrinkable behaviors are inherently associated to the optical super-oscillatory phenomenon, which is predicted and observed in optics just recently^25^,^26^,^27^,^28^. Super-oscillatory occurs in a region where a band-limited function oscillates arbitrarily quickly, faster than its highest Fourier component. Similar results have been investigated by Toraldo di Francia^29^. Our work here goes further in some aspects. Super-resolution imaging of complex patterns, instead of just focusing, is realized with the help of a relayed optics to a telescope system. This facilitates the super-resolution imaging performance for distant unmanageable targets, while confocal scanning imaging manner with active illumination fails to work. More importantly, it gives counterintuitive evidence to the common knowledge that the primary diffraction-limited images of an objective lens would not be observed in more details by the following relayed optics, as stated in the *Principles of optics*^30^.

In theory, the diffraction shrinkable spot could be arbitrarily small and yields infinitely extended bandwidth of LOTF. Unfortunately, the reduced central spot delivers polynomially decrease of central focusing energy^31^,^32^. The calculated Strehl ratios, defined as the quotient of the central intensities of a sub-diffraction spot and Airy spot, are 0.71%, 0.39% for Spot 1, Spot 2, respectively. Unlike the case in conventional imaging optics, where the reduced Strehl ratio indicates the degradation of the imaging quality mainly due to the aberrations of phase front, the ultra-small Strehl ratio deliver in this paper greatly improves the imaging resolution. Clearly, most of light energy at the focal plane is attributed to the great side lobes surrounding the viewing field. On the other hand, the ultra-low central intensity of sub-diffraction spots would impose some obstacles for light signal detection of the CCD and imaging low contrast targets. There were also some investigations reporting the super-oscillation focal spot with large Strehl ratio and small side lobes at the sacrifice of sub-diffraction spot size^33^,^34^. But the side-lobes, more than 20% of the central intensity, would seriously degrade the super-resolution imaging contrast and resolution. So one should consider the trade-off among those factors for practical applications. Regardless of the low focusing efficiency, noise influence and effects of the huge side-lobes in detecting, there seems not a theoretical limit for a super-oscillatory spot and much deeper sub-diffraction feature is possible, as indicated in some super-oscillatory investigations^32^ and illustrated in our design with 0.32 times resolvable distance of Rayleigh criterion in Supplementary Figure S2.

**Figure 4:**
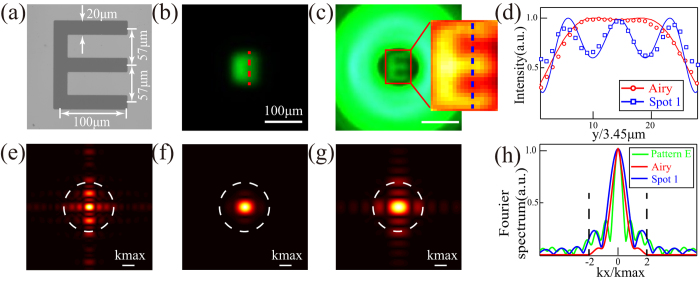
SRT imaging for a complex target. (**a**) Microscope image of an “E” target. Experimental (**b**) diffraction-limited imaging pattern and (**c**) super-resolution imaging pattern with Spot 1. Magnified false-color pattern shows the central field distribution. (**d**) Experimental (symbolic lines) and simulated (solid lines) field distributions along vertical lines in (**b,c**). (**e,f**) Simulated Fourier spectrum of (**a**,**b**,**g**) local Fourier spectrum of (**c**). (**h**) Distributions along horizontal lines across the center in (**e–g**). Dashed lines are the cut-off frequency of diffraction-limited OTF.

The SRT imaging could be obtained for complex targets in a single local field of view. As an illustrative example, the character “E” target shown in Fig. 4a is employed, which could not be resolved for diffraction-limited imaging in Fig. 4b. In the SRT imaging case shown in Fig. 4c, however, the character could be identified without ambiguity. The measured result in Fig. 4d shows good agreement with the simulation. Figure 4e–h present the calculated Fourier spectra of the character “E” target, Fourier spectra of the diffraction-limited pattern and local Fourier spectrum of the SRT imaging pattern. Clearly, the SRT system shows much higher local spatial Fourier components beyond the cut-off frequency and resembles that of “E” target in Fig. 4e.

**Figure 5:**
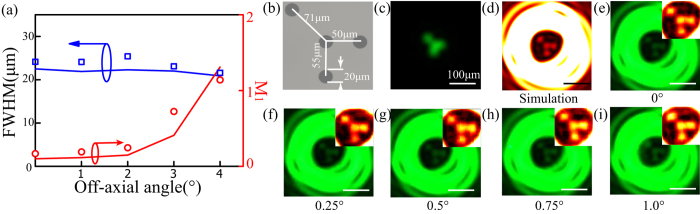
Off-axial SRT imaging. (**a**) Experimental (symbolic lines) and simulated (solid lines) FWHM and ripple level (*M*_1_) as a function of off-axis angle for Spot 1. (**b**) Microscope image of a four-hole target with variant center-to-center distances. (**c**) Experimental diffraction-limited imaging pattern and (**d**) simulated super-resolution imaging pattern with Spot 1. (**e–i**) Experimental imaging patterns for variant off-axis angles from 0° to 1° with a step of 0.25°. False-color insets show the magnified central field distributions.

It is possible to do SRT imaging of targets far away from the central optical axis, which would help imaging with a large field of view. This point could be justified by evaluating the off-axis angle dependence of the SRT’s local diffraction shrinkable behavior. As shown in Fig. 5a, both the simulated and experimental results show that the lateral full-width-at-half-maximum (FWHM) of Spot 1 hold well without apparent change for large off-axis angle up to about 3°. At the same time, the maximal relative intensity *M*_1_, which determines the imaging quality and contrast, does not increase significantly for off-axis angle smaller than 1° and after that *M*_1_ grows abruptly due to great off-axis aberration. Thus the SRT could achieve super-resolution imaging in a local field of view for a large off-axis angle. To show this point, the imaging results of the four-hole target are presented in Fig. 5e–i with off-axis angles from 0° to 1°. It could be found that the super-resolution imaging holds well for a large off-axis angle up to 1°. It is worth to note that the acceptable range of viewing field angle in SRT would be strongly dependent on the off-axis imaging aberration, which needs careful design and optimization especially for those systems with small F number. Moreover, the sub-diffraction imaging is nearly monochromatic with a narrow spectrum width in the present design of SRT. For wide light spectrum range, the inherent chromatic aberration of imaging optics and dispersion feature of phase retardation would destroy the light diffraction shrinkage behavior without further design. This concern would be relieved by the help of ultra-broadband super-oscillatory lens with plasmonic metasurfaces proposed in our previous work^22^. By combing other metasurface techniques, the bandwidth and efficiency as well as the overall performance could be further improved^35^,^36^,^37^,^38^.

**Figure 6:**
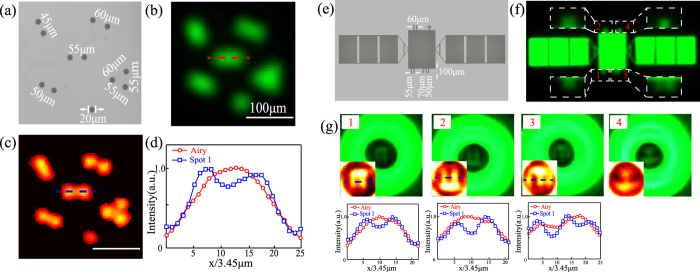
Stitched SRT imaging. (**a**,**e**) Microscope images of a group of points and a satellite-like target. (**b**,**f**) Experimental diffraction-limited imaging patterns. (**c**) Super-resolution imaging pattern with Spot 1 by superimposing together variant local fields of views. (**d**) Field distributions along horizontal lines in (**b,c**). (**g**) Local fields of views at four different positions and corresponding field distributions. False-color insets show magnified central field distributions.

For large and complex targets, a viewing field diaphragm, positioned at the diffraction-limited imaging plane of the objective lens, could be employed to avoid any distortions of huge side-lobes outside the field of view from nearby targets and enhance the overall field of view. SRT imaging could be performed by scanning the diaphragm and superimposing together multiple local fields of views at different positions. For instance, as shown in Fig. 6a–d, a group of closely positioned holes could be stitched and clearly super-resolution imaged with six local fields. We could also just focus on several parts of interest in a large target. For another pattern of a large satellite-like target (Fig. 6e), the super-resolution features like the antennas number and geometrical shape could be well discriminated through the SRT local field at four different positions (Fig. 6g).

## Discussion

It is apparent that SRT system could achieve super-resolution imaging beyond the cut-off frequency determined by the limited aperture size of the objective lens. Thus, it is reasonable to believe that diffraction-limited images of the objective lens conceal some sub-diffraction information, which is weak but optically attainable as demonstrated above. This is in coincidence with the mathematical prediction in some super-resolution restoration numerical methods proposed about fifty years ago^39^,^40^,^41^. In comparison with those methods, the presented SRT method is optically performed without the need of prior information, free from complex posterior data processing and in theory immune to incorrect restoration of ambiguous images^39^.

In brief summary, local light diffraction shrinkable phenomenon is explored and applied for real-time, on-line and incoherent SRT imaging. It is demonstrated experimentally that a SRT system could restore buried sub-diffraction information with the limited aperture size of an objective lens. Resolution as high as 0.55 times that of Rayleigh criterion is observed in experiment. In addition, experimental LPSF could hold its sub-diffraction behavior for a large off-axis angle and promises in SRT system for super-resolution imaging of complex targets by superimposing together variant local fields. In spite of the low efficiency for light focusing and time consumption to image large and complicated targets, the SRT method provides a successful way for super-resolution imaging of remote targets and promises potential applications in optical or radio astronomy observation, space surveillance and long distance optical metrics. It is believed that a relayed optics with local diffraction shrinkable effect could help those imaging devices suffering from the diffraction limit as well, like microscopes and spectroscopes.

## Methods

### Sample fabrication

The samples (diameter 8 mm) were fabricated on a glass substrate (refractive index *n* = 1.46) at an etching depth of 578 nm through reactive ion etching (RIE). The fabricating error of etching depth could be controlled under 6 nm deviation for not destroying the sub-diffraction spot (see Supplementary Information for etching tolerance).

### Measurement setup

Figure 1(a) shows the schematic of the optical measurement setup, including a halogenated lamp, a filter, an optical collimator (*L*_1_), an entrance pupil, an objective lens (*L*_2_) and a 4ƒ system consisting of a field diaphragm, two mirrors (*M*), two focusing lenses (*L*_3_ and *L*_4_), a phase plate and a CCD camera (ICL-B2520C, 2456*2058, pixel size 3.45 μm). The lenses *L*_n_ have the following focal lengths ƒ_n_: ƒ_1_ = 1000 mm, ƒ_2_ = ƒ_3_ = ƒ_4_ = 500 mm. The entrance pupil with diameter of 8 mm is positioned closely to *L*_2_. Then the exit pupil containing a phase plate and *L*_4_ should be settled at 1000 mm away from *L*_3_. The field diaphragm with diameter of 150 μm could be used for the large target scanning experiment. The narrow-band filter for the incoherent illumination centered at 532 nm has a FWHM of 10 nm. The reason for this filtering is to avoid great chromatic aberrations of the phase profile, where the diffraction shrinkage effect of LPSF is very sensitive, as could be seen in Supplementary Figure S1. As the incoherent light is incident on the 20 μm hole at the front focal plane of *L*_1_, the beam diameter incident on *L*_4_ equals 8 mm. With an effective numerical aperture *NA* = 0.008, the diffraction-limited spot on the CCD plane has a full width of 81.13 μm (1.22*λ/NA*).

### Local optical transfer function (LOTF) of SRT system

The local optical response and local optical transfer function (LOTF) are proposed and defined for the analysis of the sub-diffraction imaging behavior in the SRT system. Firstly, the point spread function in complex amplitude of SRT is divided into two parts as


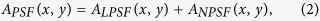


where the first part with 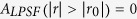 represents the shrinkage of central spot pattern beyond the diffraction limit and *r*_0_ is the radius of the field of view. Outside of the field the pattern is described by another one with 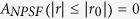. The way is in fact like wavelet transform with specially defined window^42^, which helps to analyze Fourier information within a small region of interest. Correspondingly, in coherent imaging case, the imaging pattern and its Fourier spectrum of an object function 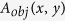 could be written as


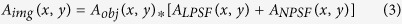



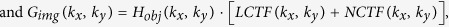


where 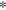 represents complex convolution operation and following functions have the Fourier relations: 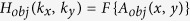, 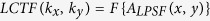 and 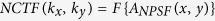.

As for incoherent imaging, the formalism below could also be proved through the relation of coherent and incoherent imaging system,


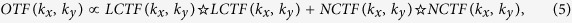


where 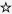 represents autocorrelation operation and 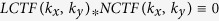. So, the limit of cut-off frequency in conventional Fourier theory of imaging optics could be readily broken from the definition of LOTF as


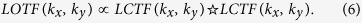


This point could be well illustrated as well in Figs 1f and 4g, where the calculated Fourier spectra for the local imaging patterns show super-resolution information beyond the cut-off frequency, defined by the limited aperture size of the objective lens.

## Additional Information

**How to cite this article**: Wang, C. *et al.* Super-resolution optical telescopes with local light diffraction shrinkage. *Sci. Rep.* **5**, 18485; doi: 10.1038/srep18485 (2015).

## Supplementary Material

Supplementary Information
