# Supplementary material for: Super-resolution optical telescopes with local light diffraction shrinkage
Source: Sci Rep. 2015 Dec 18;5:18485. doi: 10.1038/srep18485 (PMC4683440; doi:10.1038/srep18485)
Supplement: Supplementary Information [file srep18485-s1.pdf]

Supplementary Information

Super-resolution optical telescopes with local light diffraction shrinkage

Changtao Wang[1,#], Dongliang Tang[1,2,#], Yanqin Wang[1,#], Zeyu Zhao[1], Jiong Wang[1], Mingbo Pu[1], Yudong Zhang[1], Wei Yan[1], Ping Gao[1], and Xiangang Luo[1,*]

[1]State Key Laboratory of Optical Technologies on Nano-Fabrication and Micro-Engineering, Institute of Optics and Electronics, Chinese Academy of Sciences, P.O. Box 350, Chengdu 610209, China

[2]University of Chinese Academy of Sciences, Beijing 100049, China

[#]These authors contributed equally to this work.

*Correspondence and requests for materials should be addressed to X. G. L. (lxg@ioe.ac.cn)

1. Designed parameters of the spots with local light diffraction shrinkage

Table S1 describes the performance of a sub-diffraction pattern at the wavelength of 532 nm. Full width of central spot size *G* is defined as the first diameter where the intensity of the central spot falls to minimum; the field of view *L* is defined as a region where intensities of sidelobes normalized by the central peak intensity are controlled below a constant; the ripple level value M_1 is defined as the highest normalized intensity of sidelobe in the field of view, and in this work the relative value M_1 is assumed to be 0.1. The normalized π-phase-jump positions are optimized as: Airy spot with $r_1 = 0$ and $r_2 = 1$; Spot 1 with positions at $r_1 = 0.297$, $r_2 = 0.594$ and $r_3 = 0.85$; Spot 2 with positions at $r_1 = 0.4405$ and $r_2 = 0.8137$; Spot 3 with positions at $r_1 = 0.4786$ and $r_2 = 0.852$.

Parameter		Airy	Spot 1	Spot 2	Spot 3
G	S (μm)	81.13	48.68	40.57	24.34
	E (μm)	82.8	51.75	41.4	~
L	S (μm)	∞	154.15	81.13	40.57
	E (μm)	∞	151.8	79.35	~
$\boldsymbol{M_1}$	S (μm)	0.0175	0.1	0.1	0.1
	E (μm)	0.0256	0.16	0.18	~

Table S1. Simulated (S) and experimental (E) parameters for the Airy spot and other three sub-diffraction spots.

2. Rayleigh criterion for an incoherent imaging system

According to the Rayleigh criterion for incoherent illumination, the minimum resolvable distance of a two-hole target is given by $d_R = 0.61\lambda/NA$ and the intensity of midpoint is about 73.5% of the peak intensity (corresponding resolvable contrast is about 15.3%). For our work, variant distances of two-hole targets positioned at the front focal plane of the optical collimator are designed to measure the resolvable ability of SRT system. The two-hole resolvable distance of Rayleigh criterion, defined by optical collimator, is calculated to be 81.13 μm, with the effective beam diameter of 8 mm, the incident wavelength of 532 nm and the focal length of 1000 mm.

3. Chromatic and etching tolerance of sub-diffraction spots

Due to the complex interference nature of local light diffraction shrinkable spot, it is necessary to consider the chromatic aberration tolerance of the phase plate. Figure S1 plots the wavelength dependence of central sub-diffraction spot features, defined with the FWHM and peak intensity both normalized by those at the central wavelength $\lambda = 532$ nm. In our experiment, the bandwidth of illuminated wavelength is set as ±1%*λ (about ±5 nm) to keep sub-diffraction features. In addition, the etching depth tolerance error, with the same analysis of chromatic aberration tolerance, is carefully controlled as ±1% of standard etching depth (about ±6 nm) for the phase plates positioned at the exit-pupil plane of SRT system.

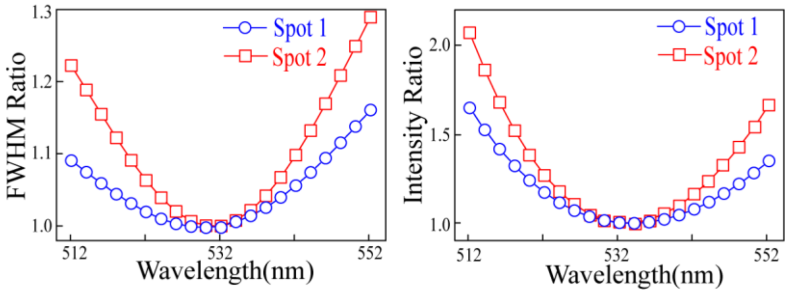

Figure S1. Chromatic aberration tolerance of sub-diffraction spots. Simulation of the FWHM ratio and intensity ratio for variant incident wavelengths.

4. Imaging simulation of Spot 3 with much deeper sub-diffraction resolution

There seems not a theoretical limit for a sub-diffraction spot and much deeper super-resolution is possible, as illustrated in Fig. S2 where two holes with center-to-center distance of 26 μm, about 0.32 times resolvable distance of Rayleigh criterion, could be resolved with Spot 3.

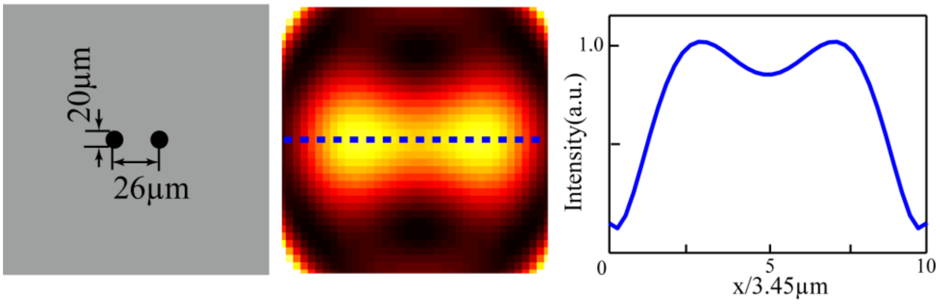

Figure S2. Resolvable ability of Spot 3. Super-resolution imaging with Spot 3 for a two-hole target with 26 μm center-to-center distance.
